# Tryptophan 2,3-Dioxygenase Expression Identified in Murine Decidual Stromal Cells Is Not Essential for Feto-Maternal Tolerance

**DOI:** 10.3389/fimmu.2020.601759

**Published:** 2020-12-08

**Authors:** Delia Hoffmann, Tereza Dvorakova, Florence Schramme, Vincent Stroobant, Benoit J. Van den Eynde

**Affiliations:** ^1^ Ludwig Institute for Cancer Research, Brussels, Belgium; ^2^ de Duve Institute, UCLouvain, Brussels, Belgium; ^3^ Walloon Excellence in Life Sciences and Biotechnology, Brussels, Belgium

**Keywords:** placenta, decidual stromal cell, immunohistochemistry, feto-maternal tolerance, TDO - tryptophan 2,3-dioxygenase, IDO1—indoleamine 2,3-dioxygenase 1

## Abstract

Indoleamine 2,3-dioxygenase 1 (IDO1) and tryptophan 2,3-dioxygenase (TDO) catalyze the rate-limiting step of tryptophan catabolism along the kynurenine pathway, which has important immuno suppressive properties, particularly in tumor cells and dendritic cells. The prominent expression of IDO1 in the placenta also suggested a role in preventing immune rejection of fetal tissues, and pharmacological inhibition of IDO1 induced abortion of allogeneic fetuses in mice. However, this was later challenged by the lack of rejection of allogeneic fetuses in IDO1-KO mice, suggesting that other mechanisms may compensate for IDO1 deficiency. Here we investigated whether TDO could contribute to feto-maternal tolerance and compensate for IDO1 deficiency in IDO1-KO mice. Expression of TDO mRNA was previously detected in placental tissues. We developed a new chimeric rabbit anti-TDO antibody to confirm TDO expression at the protein level and identify the positive cell type by immunohistochemistry in murine placenta. We observed massive TDO expression in decidual stromal cells, starting at day E3.5, peaking at day E6.5 then declining rapidly while remaining detectable until gestation end. IDO1 was also induced in decidual stromal cells, but only at a later stage of gestation when TDO expression declined. To determine whether TDO contributed to feto-maternal tolerance, we mated TDO-KO and double IDO1-TDO-KO females with allogeneic males. However, we did not observe reduced fertility. These results suggest that, despite its expression in decidual stromal cells, TDO is not a dominant mechanism of feto-maternal tolerance able to compensate for the absence of IDO1. Redundant additional mechanisms of immunosuppression likely take over in these KO mice. The massive expression of TDO during decidualization might suggest a role of TDO in angiogenesis or vessel tonicity, as previously described for IDO1.

## Introduction

Immune reactions are a fine balance between activation and suppression. Although essential for the elimination of pathogens, they can be destructive, e.g., by targeting paternal antigens in embryos. Composed of maternal and fetal cells, the placenta serves to protect and feed the embryo during pregnancy. The placenta sets up several defense mechanisms to prevent recognition of paternal antigens and immune-mediated embryo rejection (1, 2). Among others, the placenta induces the expression of enzymes that inhibit lymphocyte activity and proliferation by degrading the essential amino acid tryptophan (2).

The enzyme indoleamine 2,3-dioxygenase 1 (IDO1) catalyzes the first and rate-limiting step of tryptophan degradation along the kynurenine pathway. At the sites of IDO1 expression, the resulting tryptophan depletion, combined with the production of kynurenine and its metabolites, creates locally an immunosuppressive environment that reduces proliferation of effector T lymphocytes and favors their differentiation towards regulatory T lymphocytes (3–5). IDO1 expression is strongly induced by interferon gamma (IFNγ) in inflammatory lesions, where it appears to prevent immunopathology by negatively regulating immune responses (6–9). Under non-inflammatory conditions, IDO1 is expressed in scattered cells in the lung, female genital tract and secondary lymphoid organs (6). It is also highly expressed in placenta and tumors (6), which were shown to benefit from its immunosuppressive function (10, 11). In the human placenta, IDO1 is expressed in endothelial cells, syncytiotrophoblasts, extravillous trophoblasts, stromal cells, and macrophages (6, 12–14). The murine placenta, where *Ido1* mRNA is present between embryonic ages E8.5 and E12.5 (15), is the first organ where IDO1 was associated with immunosuppression. Upon pharmacological inhibition of IDO1, mice rejected allogeneic, but not syngeneic, concepti in an immune-dependent manner (10). A large number of human tumors, including endometrial, cervical, renal, non-small cell lung and colorectal carcinomas, express IDO1 (6, 11). IDO1 expression in tumor cells is either induced by lymphocyte-derived IFNγ (16) or expressed by an autocrine feedback loop driven by COX2 (17). IDO1 can also be expressed by other cells in the tumor microenvironment, including dendritic cells and vascular endothelial cells (6, 11). IDO1 expression favors tumoral resistance to immune rejection (11, 18–21). These results led to the development of IDO1 inhibitors that are currently in clinical development (22).

In contrast to pharmacological inhibition of IDO1, IDO1-KO females failed to reject allogeneic concepti (23). The authors hypothesized that feto-maternal tolerance may be rescued in these mice by tryptophan 2,3-dioxygenase (TDO encoded by *Tdo2*), the second enzyme initiating tryptophan to kynurenine degradation. TDO is expressed under normal physiological conditions in the liver to stabilize the systemic tryptophan concentration by degrading excess dietary tryptophan (24, 25) and in the brain where it might produce neuroactive compounds (26). In pathology, TDO is expressed by tumor cells of all hepatocarcinomas and 25% of glioblastomas, as well as by pericytes of vascular structures in focal spots of most late stage tumors, and by pericytes in pulmonary inflammatory lesions (27, 28). TDO inhibition of either tumoral or hepatic TDO favors immune-mediated tumor rejection and increases the efficacy of immune checkpoint inhibitors (29, 30). TDO was also identified in pericytes and interstitial syncytiotrophoblasts in the human placenta (27). Although its mRNA was found in the murine decidua during early pregnancy (15, 31, 32), the cell type expressing TDO was not yet identified due to the lack of specific monoclonal antibodies. By analogy to placental IDO1, it is believed that TDO may contribute to feto-maternal tolerance and may rescue placental immunosuppression in the absence of IDO1 (23), but this hypothesis remained to be tested.

In this report, we produced a chimeric rabbit anti-TDO antibody and characterized the expression of TDO in the murine placenta throughout pregnancy. We also tested the role of TDO and IDO1 in feto-maternal tolerance, by mating TDO-KO and double IDO1-TDO-KO females with syngeneic and allogeneic males.

## Materials and Methods

### Mice

C57BL/6J Ola Hsd mice (abbreviated B/6) were purchased from Envigo. B/6 TDO-KO mice were kindly provided by Dr Hiroshi Funakoshi and Dr Toshikazu Nakamura from Osaka University (24). B/6 and B/6 TDO-KO mice were mated to obtain heterozygous offspring, which were intercrossed to obtain *Tdo2* ^+/+^ (WT), *Tdo2* ^+/-^, and *Tdo2* ^-/-^ (TDO-KO) mice. Homozygous WT and TDO-KO littermates were selected by PCR on genomic DNA. B6.129 IDO1_tm1Alm_/J (abbreviated B/6 IDO1-KO) mice were purchased from Jackson Laboratory. B/6 and B/6 IDO1-KO mice were mated to obtain heterozygous offspring, which were intercrossed to obtain *Ido1* ^+/+^ (WT), *Ido1* ^+/-^, and *Ido1* ^-/-^ (IDO1-KO) mice. Homozygous WT and IDO1-KO littermates were selected by PCR on genomic DNA. B/6 TDO-KO and B/6 IDO1-KO mice were mated to obtain double IDO1-TDO-KO mice and were described in our previous publication (30). CBA/Ca Ola Hsd mice (abbreviated CBA) were purchased from Envigo. BALB/c IDO1-KO mice were a gift from P. Matthys, KULeuven, Rega Institute, Leuven, Belgium and were originally described by Baban and co-workers (23). DBA/2 Ola Hsd mice were purchased from Harlan Laboratories. Mice were bred at the animal facility of the Ludwig Institute for Cancer Research, Brussels, Belgium and were used at 12–18 weeks of age. Animal studies were conducted in accordance with national and institutional guidelines for animal care and with the approval of the Comité d’Ethique pour l’Expérimentation Animale from the Secteur des Sciences de la Santé, UCLouvain (2011/UCL/MD/015 and 2015/UCL/MD/015).

The following primers were used for genotyping (F = forward, R = reverse):

m*Tdo2*:

F 5’-GTATCTATGGAGGACAATGAAG-3’
R 5’-GATGAATAGGTGCTCGTCATG-3’
*Neomycin resistance gene* replacing *Tdo2*:
F 5’-GTTCTTTTTGTCAAGACCGA-3’
R 5’-TTTCCACCATGATATTCGGC-3’.
m*Ido1*:
F 5’-TGGAGCTGCCCGACGC-3’
R 5’-TACCTTCCGAGCCCAGACAC-3’
*Neomycin resistance gene* replacing *Ido1*:
F 5’-CTTGGGTGGAGAGGCTATTC-3’
R 5’-AGGTGAGATGACAGGAGATC-3’.

### Cell Lines

HEK-293-EBNA (293E) cells were purchased from InvivoGen, were tested for *Mycoplasma* in October 2019 and were authenticated in November 2019 by short tandem repeat profiling (Promega Powerplex hs 16). P815B were a gift from P. Chen, Harvard Medical School. P815B transfected with m*Tdo2* were described previously (29). We used the P815B-mTDO clone 12. They were not authenticated and not tested for *Mycoplasma* in the past year.

### Chimeric Rabbit Anti-TDO Antibody Production

cDNA was prepared from the mouse anti-TDO clone V hybridoma (27). The variable region of the heavy chain was amplified using the following primers: 5’-AGACACTGAATCTCAAGGTC-3’ (forward), 5’-GCTGAGGAGACTGTGAGAGT-3’ (reverse). The PCR product was cloned in the pFUSE-rIgG-Fc1 vector (InvivoGen, #pfuse-rfc1) containing the constant region of the rabbit heavy chain. The variable region of the light chain was amplified using the following primers: 5’-AGACAGGCAGTGGGAGCAAG-3’ (forward), 5’-GCCCGTTTTATTTCCAGGTT-3’ (reverse). The PCR product was cloned in the pFUSE2-CLIg-rk1 vector (InvivoGen, #pfuse2-rclk1) containing the constant region of the rabbit kappa 1 light chain. 293E cells were transfected with both vectors by electroporation and the cells were selected with 5 µg/ml of blasticidin and with 400 µg/ml of zeocin. The rabbit antibody was purified from the cell culture supernatant with a HiTrap Protein G HP column (GE Healthcare).

### Tissue Preparation for Antibody Validation

Livers were dissected from B/6 WT and TDO-KO mice. 200,000 untransfected P815B cells or P815B-mTDO clone 12 cells were injected subcutaneously in DBA/2 mice. The tumors were dissected after 17–28 days. The tissues were fixed overnight in 4% formaldehyde at 4°C and embedded in paraffin using the Vacuum Infiltration Processor (Tissue-Tek).

### Matings and Tissue Processing

The males were isolated for 1 week and then mated for 1 week as preparation. After this training period, the experiments were started. For experiments where the gestation outcome was monitored, the mice were mated in bigamous couples, the females were separated from the males after 6 days, isolated 17 days after mating and the gestation outcome was monitored by counting the number of females giving birth and the number of pups per female. For experiments where the females were dissected between the embryonic ages E0.5 and E17.5, couples were made with 2-4 females per male and vaginal plugs were checked every morning before 9 AM. The morning when a plug was detected became day E0.5. The uteri of females dissected between E0.5 and E9.5 were entirely processed. A part was frozen for RNA extraction and the remaining tissue was fixed over-night in 4% formaldehyde at 4°C. Between E10.5 and E17.5, the embryos were discarded and the placenta and uterus were separately frozen. The remaining tissue was entirely fixed. The fixed tissues were embedded in paraffin using the Vacuum Infiltration Processor (Tissue-Tek).

### Serum Sampling and Kynurenine Quantification

Three drops of blood were collected by tail vein bleeding, coagulation took place during 1 h at room temperature and serum was removed after centrifugation. Serum kynurenine was quantiﬁed by HPLC based on the retention time and the UV absorption (360 nm) (29).

### Quantitative RT-PCR (RT-qPCR)

Frozen tissues were crushed in the lysis buffer of the RNA extraction kit using a TissueLyser LT (Qiagen). RNA was then extracted with NucleoSpin RNA (Macherey Nagel) according to the manufacturer’s instructions. RNA was quantiﬁed with a NanoDrop spectrophotometer and a deﬁned amount of RNA was retrotranscribed by the RevertAid RT Kit (Thermo Fisher Scientiﬁc). TaqMan qPCR was performed with Takyon ROX Probe 2X MasterMix dTTP blue (Eurogentec) in a StepOnePlus thermal cycler (Applied Biosystems) using the following program for murine *Ido1* and *Tdo2*: 3 min at 95°C, then 40 cycles of 10 s at 95°C and 1 min at 60°C; for murine *β-actin (Actb)*: 3 min at 95°C, then 40 cycles of 3 s at 95°C and 30 s at 60°C. The following primers were used (F = forward, R = reverse, P = probe):

m*Ido1*:

F 5’-GTACATCACCATGGCGTATG-3’
R 5’-CGAGGAAGAAGCCCTTGTC-3’
P 5’-CTGCCCCGCAATATTGCTGTTCCCTAC-3’
m*Tdo2*:
F 5’-GTATCTATGGAGGACAATGAAG-3’
R 5’-GATGAATAGGTGCTCGTCATG-3’
P 5’-CCTCCTTTGCTGGCTCTGTTTACACC-3’
m*β-actin (Actb)*:
F 5’-CTCTGGCTCCTAGCACCATGAAG-3’
R 5’-GCTGGAAGGTGGACAGTGAG-3’
P 5’-ATCGGTGGCTCCATCCTGGC-3’

The probes were coupled to 5’ FAM and 3’ TAMRA. Standard curves were added for each gene. m*β-actin* was used for normalization.

### Immunohistochemistry (IHC) and Immunofluorescence (IF) Multiplex Stainings

Paraffin sections (5 µm thick) were deparaffinized in three baths of Histo-Clear (3 min each), washed in butanol for 3 min and progressively rehydrated in 100, 90, 70, and 50% ethanol and in demineralized water (3 min each). Antigen retrieval was performed with Tris/EDTA buffer at pH 9 (in case of TDO IHC and IDO1-TDO IF) or with citrate buffer at pH 6 (in case of IDO1 IHC and IDO1-PDGFRβ IF) in a 2100 Antigen Retriever (Aptum) using a pre-defined heating cycle. All the following steps were performed at room temperature. Endogenous peroxidases were blocked with Peroxidase Block (Dako) for 15 min and protein blocking was done for 1 h with TBS-Tween containing 2% milk, 5% bovine serum albumin (biotin-free BSA) and 1% human IgG. The primary antibodies were diluted in IHC diluent (Enzo) and incubated for 1 h. The chimeric rabbit anti-TDO antibody was used at 5 µg/ml. Rabbit anti-IDO1 clone D8W5E (Cell Signaling Technology, #51851S) was used at 1:5,000. Rabbit anti-PDGFRβ clone Y92 (Abcam, #ab32570) was used at 1:200. After washing, the secondary antibody EnVision+ HRP goat anti-rabbit (Dako, #K4003) was incubated for 1 h. For IHC, the staining was revealed with HIGHDEF DAB substrate (Enzo) or with AEC+ Substrate Chromogen (Dako) between 5 and 20 min and counterstaining was performed with hematoxylin. Slides were incubated for 5 min in hematoxylin, washed with demineralized water, washed in tap water for 5 min and were transferred again to demineralized water. Slides were mounted with HIGHDEF IHC mount (Enzo). For multiplex IF, the staining was revealed with the Tyramide Signal Amplification (TSA) system. Alexa Fluor (AF) 488 and 647 tyramide reagents (ThermoFisher Scientific) were used at 1:50 in borate buffer. The antibodies were then eluted with citrate buffer at pH 6 using microwave treatment at 900 W until boiling followed by 15 min at 90 W. The following staining was started with protein blocking and performed as described above. After the second staining, microwave treatment was performed, the nuclei were counterstained for 5 min with Hoechst 33342 (Invitrogen) used at 20 µg/ml in 0.1% TBS-Tween with 10% BSA, and the slides were mounted with HIGHDEF IHC fluoromount (Enzo). The slides were digitalized using a Pannoramic 250 Flash III tissue scanner (3DHISTECH) at ×20 magnification.

### Statistics

Statistical analyses were performed using Prism 6 (GraphPad Software).

## Results

### Production and Validation of a Chimeric Rabbit Anti-TDO Antibody

Given the lack of antibodies suitable for IHC stainings of murine TDO, we first produced a rabbit antibody derived from the murine TDO-specific mAb V, with was previously found to recognize murine TDO as a single band by western blot (27). To avoid detection of endogenous IgG in murine tissues with the secondary antibodies, we replaced the Fc portion of mAb V with that of a rabbit IgG (**Figure S1A**). Used in IHC, the chimeric rabbit TDO mAb V stained the liver of WT, but not TDO-KO mice, and stained TDO-transfected but not TDO-negative P815B tumors (**Figure S1B**).

### Expression Profile of TDO in the Murine Placenta

**Figure 1:**
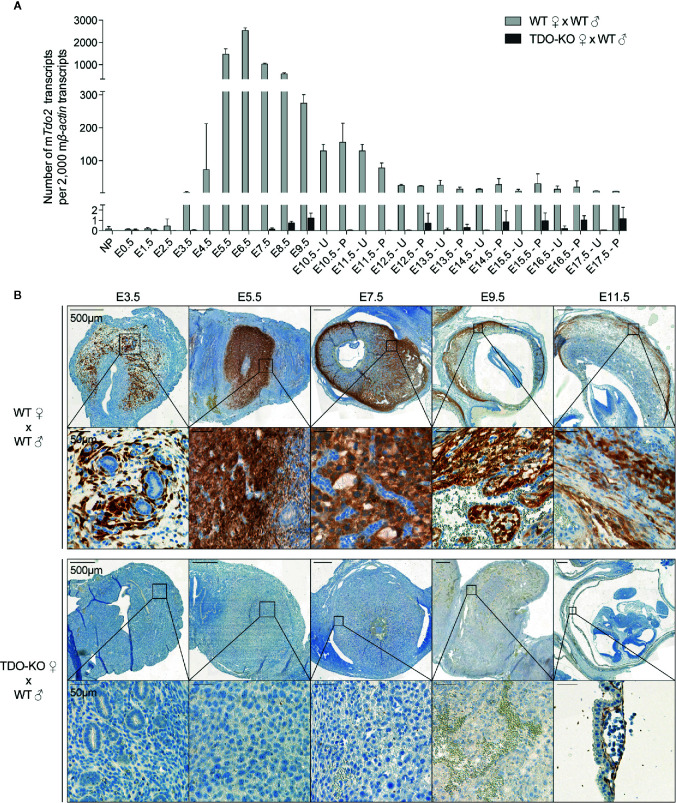
TDO expression kinetics in the maternal part of the mouse placenta (decidua). **(A)** WT and TDO-KO B/6 females were mated with syngeneic WT B/6 males and RNA was extracted from uteri with concepti between days E0.5 and E9.5 of gestation and separately from placenta and uteri between days E10.5 and E17.5 of gestation (2–10 females/group). *Tdo2* expression was measured by RT-qPCR and reported to 2,000 copies of murine *β-actin* (mean ± SD). **(B)** Sections from the same tissues were stained for TDO by IHC using the chimeric TDO mAb V. Negative controls were performed by omitting the primary antibody and remained unstained. NP, not pregnant; E, embryonic age; U, uterus; P, placenta.

**Figure 2:**
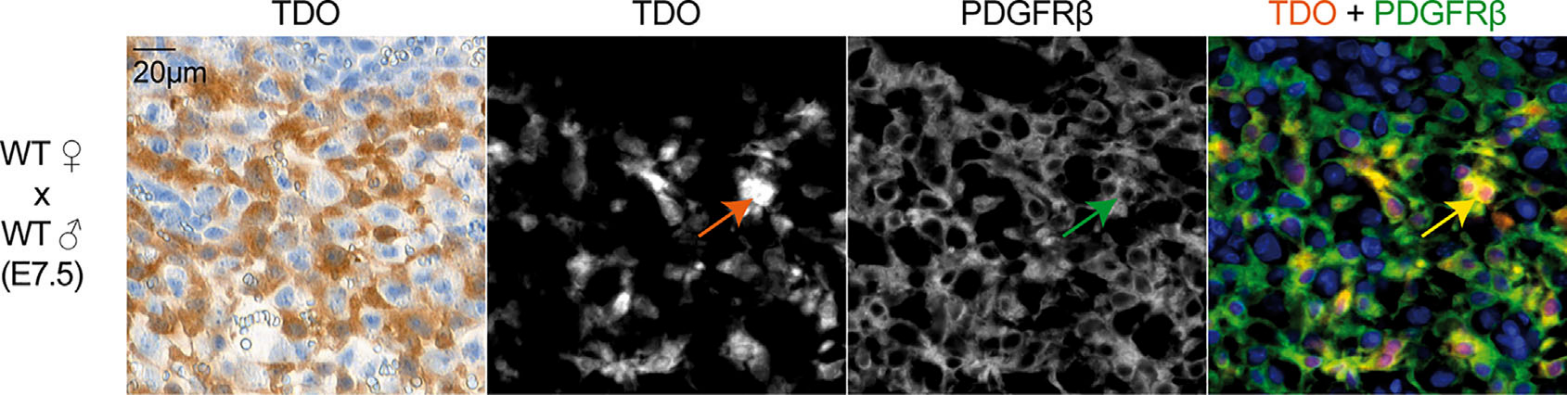
TDO expression by decidual stromal cells. IHC (panel 1) and immunofluorescence co-staining (panels 2–4) on adjacent tissue sections of murine uterus at gestation age E7.5 (WT female mated with WT male). IHC was performed using the chimeric TDO mAb V. Immunofluorescence was performed for TDO using the chimeric TDO mAb V (orange, AF647) and PDGFRβ (green, AF488). Co-localization of both markers appears in yellow. The arrows point a cell that co-expresses TDO and PDGFRβ. Negative controls were performed by omitting the primary antibodies and remained unstained.

We first used RT-qPCR to characterize the kinetics of *Tdo2* expression in decidual and placental tissues from WT and TDO-KO gravid female mice mated with WT males. We confirmed *Tdo2* expression in the early phases of gestation (15, 31), starting around day E3.5, peaking at day E6.5 and then contracting progressively until day E12.5, when it reached a basal level that remained stable until the gestation end (**Figure 1A**). This *Tdo2* expression was absent in placenta from TDO-KO females, indicating that it was mostly contributed by maternal tissues. IHC stainings of TDO with our chimeric rabbit antibody on the same samples confirmed the RT-qPCR results, with a massive TDO expression observed around days E5.5 and E7.5 and localized in the decidualized endometrium, which is the maternal part of the placenta (**Figure 1B**). TDO-expressing cells were absent from tissues from TDO-KO females, except for one layer of cells in the amniotic sac, which were visible from day E11.5, and presumably expressed the paternal *Tdo2* allele, as the amniotic sac derives from the embryo (**Figure 1B**). This likely explains the low *Tdo2* expression detected by RT-qPCR in late gestation stages in TDO-KO females (**Figure 1A**). Of note, the low *Tdo2* expression observed by RT-qPCR around day E9.5 in TDO-KO females may reflect the paternal allele expressed by the developing fetal liver. At later time points, the embryos were carefully dissected out of the placental tissues so that the fetal liver no longer contaminated the samples. Co-stainings of TDO with the beta-type platelet-derived growth factor receptor (PDGFRβ) indicated that the TDO-expressing cells corresponded to decidual stromal cells (**Figure 2**). This differed from the expression pattern in the human placenta where we reported TDO expression in fetal pericytes and in interstitial trophoblasts (27).

### TDO Increases Serum Kynurenine Concentration During Pregnancy

**Figure 3:**
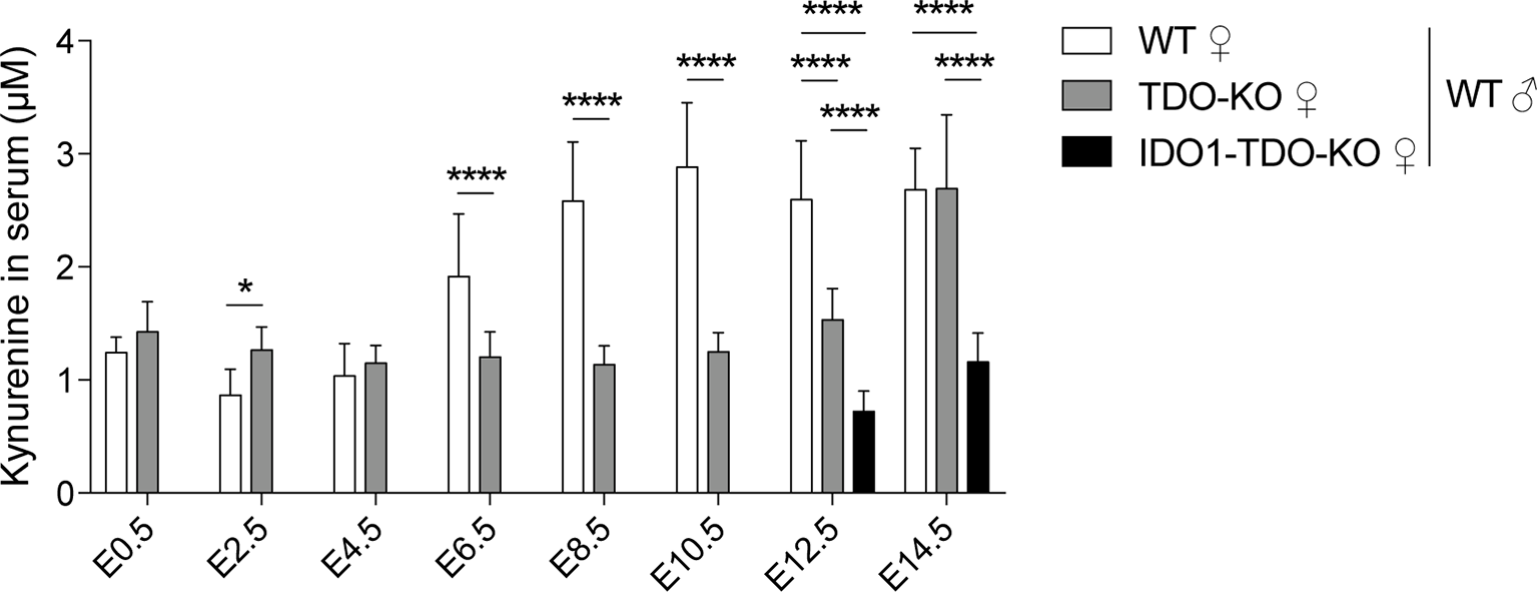
Serum level of kynurenine in gravid mice. Kynurenine was measured in gravid WT, TDO-KO and IDO1-TDO-KO B/6 females mated with WT B/6 males. Blood samples were taken every 2 days between days E0.5 and E14.5 of gestation (mean ± SD, 9–11 females/group, two-way ANOVA with Tukey’s multiple comparisons test: *P < 0.0332; ****P < 0.0001). E, embryonic age.

We studied the enzymatic activity of TDO by measuring the serum concentration of the downstream metabolite kynurenine. We observed that the kynurenine concentration in WT females increased during pregnancy concomitantly with TDO induction in the placenta, starting around E6.5 and peaking at E10.5 (**Figure 3**). This increase during early pregnancy was abolished in TDO-KO and double-KO (IDO1-TDO-KO) females mated with WT males (**Figure 3**). These results indicated that TDO expressed by decidual cells was metabolically active and secreted high amounts of kynurenine into the bloodstream.

### Role of Placental TDO in Feto-Maternal Tolerance

**Figure 4:**
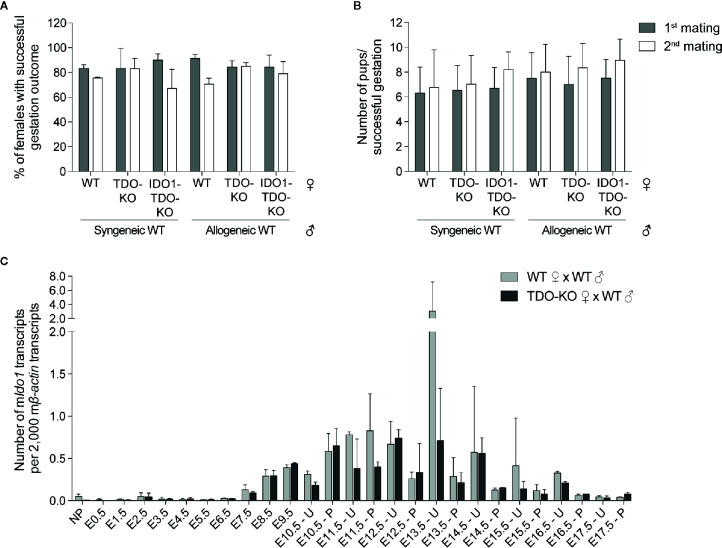
Outcome of gestation in TDO-KO and double IDO1-TDO-KO females. **(A, B)** WT littermate, TDO-KO littermate or IDO1-TDO-KO B/6 females were mated with either syngeneic WT B/6 males or allogeneic WT CBA males (20 females/group). The number of females giving birth and the number of pups per successful gestation were counted (gray bars). The females that gave birth were mated a second time with the same males (white bars). **(A)** Percentage of females giving birth (mean ± SD of 3 (1st mating) or 2 (2nd mating) independent experiments). **(B)** Numbers of pups per successful gestation [mean ± SD of pups pooled from 3 (1st mating) or 2 (2nd mating) independent experiments]. **(C)** WT and TDO-KO B/6 females were mated with syngeneic WT B/6 males and RNA was extracted from uteri with concepti between days E0.5 and E9.5 of gestation and separately from placenta and uteri between days E10.5 and E17.5 of gestation (2–4 females/group, the same samples as in **Figure 1A**). *Ido1* expression was measured by RT-qPCR and reported to 2,000 copies of murine *β-actin* (mean ± SD).

Expression of IDO1 was shown to contribute to feto-maternal immune tolerance by suppressing alloreactive T lymphocytes (10). This was based on the observation that the IDO1 inhibitor 1-methyl tryptophan (1MT) induced immune-mediated abortion of allogeneic concepti (10). Yet, subsequent studies showed that feto-maternal tolerance was maintained in IDO1-KO mice (23). One potential explanation for this was a compensatory mechanism by placental TDO in IDO1-KO mice (23). We therefore explored the potential role of placental TDO in feto-maternal tolerance. We crossed WT and TDO-KO B/6 females (H-2^b^) with syngeneic B/6 males or allogeneic CBA males (H-2^k^). We observed no difference in the number of females giving birth (**Figure 4A**), nor in the number of pups per female giving birth (**Figure 4B**). We then mated a second time the females who had a successful gestation outcome after the first mating, to see if the females needed a first contact with the paternal antigens before mounting an immune reaction against the embryo. We observed no difference either (**Figures 4A, B**). We therefore hypothesized that IDO1 may rescue the absence TDO.

### Expression Profile of IDO1 in the Murine Placenta

**Figure 5:**
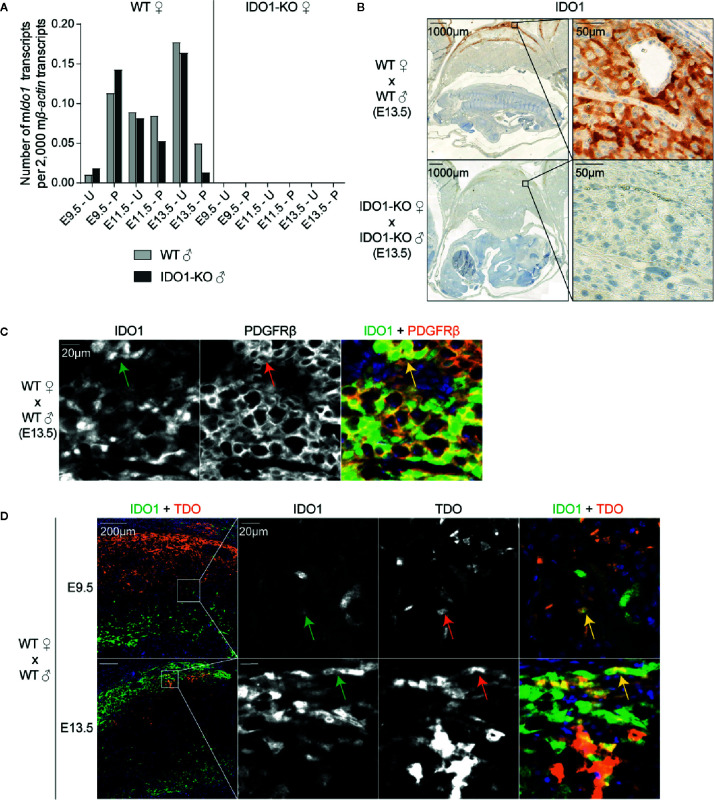
IDO1 expression by stromal cells in the decidua. **(A)** WT and IDO1-KO B/6 females were mated with syngeneic WT or IDO1-KO B/6 males and RNA was extracted separately from placenta and uteri between days E9.5 and E13.5 of gestation. *Ido1* expression was measured by RT-qPCR and reported to 2,000 copies of murine *β-actin* (mean ± SD). **(B)** Corresponding FFPE tissues (day E13.5) were stained for IDO1. **(C)** Immunofluorescence co-staining of IDO1 (green, AF488) and PDGFRβ (orange, AF647) were performed on a FFPE tissue section of a WT uterus (day E13.5). The arrows point cells that co-express IDO1 and PDGFRβ. **(D)** Immunofluorescence co-staining of TDO (orange, AF647) and IDO1 (green, AF488) was performed on FFPE tissue sections of WT uteri at E9.5 and E13.5. Co-localization of both markers appears in yellow. The arrows point cells that co-express TDO and IDO1. Negative controls were performed by omitting the primary antibodies and remained unstained. E, embryonic age; U, uterus; P, placenta.

IDO1 expression was induced around day E8.5, at the time when TDO expression started decreasing (**Figures 4C** and **5A**) (15). The complete absence of *Ido1* mRNA in placenta from IDO1-KO females mated with WT males indicated that *Ido1* expression exclusively depended on the maternal allele (**Figure 5A**) (23). IHC stains confirmed that IDO1 was expressed in the maternal part of the WT placenta (i.e. the decidua) while IDO1-KO placentas remained unstained (**Figure 5B**). Co-stainings with PDGFRβ showed that IDO1-expressing cells were decidual stromal cells, like TDO-expressing cells (**Figure 5C**). Co-stainings for IDO1 and TDO indicated that cells expressing either enzyme were usually located in different parts of the tissue, although some cells located at the interface between these areas co-expressed both enzymes (**Figure 5D**).

### IDO1 Does Not Rescue Feto-Maternal Tolerance in the Absence of TDO

Because it was possible that IDO1 compensated for the lack of TDO in TDO-KO females, we also crossed double knockout IDO1-TDO-KO females with WT allogeneic males and assessed pregnancy outcome. However, IDO1-TDO-KO females were as fertile as WT females (**Figures 4A, B**), indicating that IDO1 did not rescue feto-maternal tolerance in the absence of TDO. To confirm this observation, we studied *Ido1* expression by RT-qPCR in uteri and placentas from WT and TDO-KO mice at different time-points of pregnancy. We did not see more *Ido1* expression in TDO-KO females as compared to WT mice, indicating the absence of compensatory overexpression of *Ido1* in these mice (**Figure 4C**).

To further exclude a potential role of the paternal IDO1, we also mated WT, IDO1-KO and double knockout IDO1-TDO-KO females with WT or IDO1-KO males. We observed no difference in terms of number of successful gestations and number of pups per gestation (**Figures S2A, B**). This was not surprising, because IDO1 expression in the placenta was found exclusively in maternal tissues (**Figures 5A, B**) (23). We also did not see more *Tdo2* expression in IDO1-KO females to rescue the lack of IDO1 (**Figure S2C**). Our results thus confirmed that IDO1-KO mice resist immune-mediated embryo rejection (23), despite initial data showing that pharmacological inhibition of IDO1 with 1MT specifically affected allogeneic pregnancies (10). We then repeated this initial experiment by treating allogeneically and syngeneically crossed WT females with 1MT and evaluating fertility at E15.5, the time-point by which all allogeneic concepti were reported to be rejected (10). Surprisingly, we did not observe a worsened outcome in 1MT-treated allogeneic pregnancies (**Figure S3A**), although the subcutaneously implanted 1MT pellets released well the inhibitor in the blood (**Figure S3B**).

Altogether, we observed expression of TDO and IDO1 in murine decidual stromal cells; TDO was induced during early pregnancy at the time of decidualization, causing an increase in circulating kynurenine, and was replaced during mid-gestation by IDO1. Using single and double KO mice, we could not confirm an essential role for these enzymes in feto-maternal tolerance.

## Discussion

The role of IDO1 in feto-maternal tolerance was initially suggested by the observation that pharmacological inhibition of IDO1 with 1MT in females mated with allogeneic males induced immune-mediated abortion (10). However, subsequent studies from the same group showed that allogeneic concepti were not rejected by IDO1-KO mice (23). The authors had two hypotheses to explain these discrepancies. First, they suggested that 1MT could have off-target effects, but they rejected this hypothesis by showing that 1MT treatment of allogeneically pregnant IDO1-KO females did not reduce pregnancy success rate (23). Second, they hypothesized that other immunosuppressive mechanisms, resistant to 1MT, could rescue the lack of IDO1 in KO mice (23). They suggested TDO as a compensatory mechanism, because it is expressed at the maternal-fetal interface and it exerts an enzymatic activity similar to that of IDO1 except for the fact that it is not blocked by 1MT. To test this hypothesis, we performed syngeneic and allogeneic crossings with IDO1-KO, TDO-KO, and double IDO1-TDO-KO mice. We showed that neither single enzyme deficiency, nor the combined loss of IDO1 and TDO worsened allogeneic pregnancy outcome (**Figure 4** and **Figure S2**). We therefore concluded that TDO did not compensate for the lack of IDO1, at least not in a dominant manner, and that additional immunosuppressive mechanisms likely take over to ensure feto-maternal tolerance in these genetically deficient mice (1, 2).

To further clarify the discrepancies between IDO1-KO and 1MT-treated mice, we repeated the key initial experiment performed by Munn and co-workers, and treated WT allogeneically pregnant mice with 1MT. Although we used identical experimental conditions, we did not observe rejection of allogeneic concepti upon implantation of subcutaneous 1MT pellets (**Figure S3A**). The reason for this different outcome is unclear. We verified proper release of 1MT by measuring 1MT in the blood. Of note, the surgery performed to implant the pellets was stressful for the mice and harmful for pregnancy outcome. Under normal treatment and housing conditions, we observed that about 70% of the females with a vaginal plug were actually fertilized, but this success rate was reduced to 40% in females that underwent surgery at E4.5 (**Figure S3A**). Surprisingly, allogeneically mated females treated with 1MT showed the highest success rate because 7 out of 9 treated females (78%) contained concepti at E15.5, although the opposite result was expected (**Figure S3A**). It should be considered for future studies that surgery is a stressful procedure that should be replaced by another route of inhibitor administration. New IDO1 inhibitors have been developed that are more potent than 1MT and can be delivered by the oral route (33–35). Such experiments would be needed to conclude whether or not tryptophan catabolism plays a role in feto-maternal tolerance.

In this study, we also provide a detailed characterization of TDO and IDO1 expression in the murine uterus and placenta during pregnancy not only at the transcript level but also at the protein level using a novel chimeric rabbit TDO-specific antibody that we used for IHC. We observed that both proteins were expressed by endometrial stromal cells during the decidualization process, but with different kinetics and in different tissue regions. TDO was induced at early gestation time points, peaked around E6.5 (**Figure 1**) (15, 31, 32) and was replaced by IDO1 at later time points (**Figures 4C** and **5A**) (15). Of note, human and murine placentas differ with regard to the type, origin, and abundance of TDO-positive cells. In the human decidua, TDO is expressed by some rare scattered cells, which are all of embryonic origin and correspond either to pericytes inside placental villi or to interstitial trophoblasts (27). In mice, TDO is massively expressed by maternal endometrial stromal cells during decidualization (**Figure 1**). The cell type expressing IDO1 in the mouse placenta was not yet clearly identified. Different studies using polyclonal anti-murine IDO1 antibodies revealed IHC staining in maternal and fetal cells, notably in endometrial epithelial and scattered stromal cells, trophoblast giant cells, syncytial cells of the labyrinth and cells of the chorionic membrane (23, 36). We used a monoclonal antibody, validated by IHC on WT and IDO1-KO placenta, and showed that IDO1 was expressed by decidual stromal cells concomitantly with the presence of *Ido1* mRNA in the same tissues (**Figure 5B**). This is different from the human placenta, which was previously described to express IDO1 in endothelial cells (6). We concluded that both IDO1 and TDO expression patterns showed distinctive inter-species differences. Due to these major discrepancies, it might be difficult to translate results obtained in mice to humans.

As an indication of the enzymatic activity of IDO1 and TDO, we measured kynurenine in the serum of pregnant mice (**Figure 3**). We showed in a previous report that circulating kynurenine in non-pregnant mice was produced by IDO1 and not by hepatic TDO, as it was completely absent in IDO1-KO mice, while it was slightly increased in TDO-KO mice in which tryptophan levels were elevated (30). We confirmed this result at early pregnancy time points, when serum kynurenine in TDO-KO mice was identical or slightly higher than in WT mice, while it was not detectable in double IDO1-TDO-KO mice. However, kynurenine increased in the serum after E6.5, concomitantly with TDO induction in the placenta. This increase indicated enzymatic activity of placental TDO, as it was not observed in TDO-KO mice and was not rescued at day E8.5, when IDO1 was induced. Kynurenine levels only increased after E12.5 in single and double KO mice, perhaps due to the development of the fetal liver expressing paternal-derived TDO. In humans, the kynurenine to tryptophan ratio increases progressively between the first and the third trimester, which is for the most part due to a decrease in seric tryptophan (37). Kynurenine concentrations are only mildly affected during pregnancy. After an initial decrease, kynurenine slightly increases in the serum between the second and the third trimester, but to a much smaller extent compared to mice (38). The aforementioned interspecies discrepancies in the expression profiles of IDO1 and TDO therefore seem to result in different metabolite profiles in human and murine serum. Of note, kynurenine and tryptophan concentrations vary according to the sampling site, and both concentrations are higher in human umbilical cord vein serum compared to maternal serum (39). Serum concentrations have to be considered with care because they are only indirect indications of the metabolic activity of TDO and IDO1. First, the metabolites are highly diluted in the serum. Second, kynurenine levels in the serum also depend on the presence of downstream enzymes that further degrade kynurenine along the kynurenine pathway. The expression of such enzymes in the liver likely explains why hepatic TDO is not responsible for circulating kynurenine levels. In humans, first trimester and term placentas were shown to express downstream enzymes of the kynurenine pathway (13, 40, 41). Third, serum gives no information about the origin of metabolites. It does not distinguish hepatic from placental TDO, or placental from non-placental IDO1 (e.g., expressed in the lungs, intestines, or lymph nodes). To clarify these points, it would be useful to analyze the interstitial fluid (30) or the umbilical cord serum (39) of placenta-specific KO mice.

In view of the open questions about their role in feto-maternal immune tolerance, the specific expression of IDO1 and TDO in placenta might indicate an additional physiological role of IDO1 and TDO in this tissue. Recent studies in IDO1-KO mice have highlighted a role for IDO1 in supporting neovascularization, particularly in inflammatory settings including oxygen-induced retinopathy and cancer (42, 43). IDO1-KO mice also display reduced normal pulmonary vascularization (43). Upon inflammatory conditions, vessel tonicity can be regulated by IDO1 and its metabolites (44). Not only kynurenine but also a newly described tryptophan-derived tricyclic hydroperoxide formed by IDO1 in the presence of H_2_O_2_ were described to cause vessel relaxation, and may contribute to the pathogenesis of septic shock (44, 45). Increasing evidence also suggests a role for IDO1 in placental vessel relaxation and an implication in the pathophysiology of pre-eclampsia, a disease which is characterized amongst others by maternal hypertension. IDO1-deficient mice develop pre-eclampsia-like symptoms during gestation, such as mildly elevated blood pressure (46). Tryptophan metabolized by IDO1 causes vessel relaxation in explants from human placenta (47, 48). Placentas from patients with pre-eclampsia, intrauterine growth restriction or recurrent miscarriage show reduced expression and enzymatic activity of IDO1 (47, 49, 50). In another study, reduced IDO1 expression in villous stromal endothelial cells of human placentas with pre-eclampsia correlated with more severe maternal hypertension and proteinuria (51). Since TDO catalyzes the same enzymatic reaction as IDO1, it is tempting to speculate that TDO might also play a role in neovascularization. Several observations support this hypothesis. In human samples, we observed TDO-expressing pericytes in areas characterized by neoangiogenesis, such as in inflammatory granulomas (52), or in hemorrhagic or necrotic tumor areas (27). The cytokine IL-1β, which increases TDO expression in primary cultures of human endometrioma stromal cells, also promotes an angiogenic phenotype in these cells (53, 54). The expression of TDO is reduced in placentas from patients showing fetal growth restriction (39), although it is increased in patients with pre-eclampsia possibly as a compensation for reduced IDO1 expression (49). In mouse placenta, the burst of TDO expression we report here in decidual stromal cells is concomitant with high angiogenesis in the placenta (55, 56). Although only correlative, this observation is also in line with a possible role of TDO in angiogenesis, which is worthy of further investigations.

## Data Availability Statement

The original contributions presented in the study are included in the article/**Supplementary Material**. Further inquiries can be directed to the corresponding author.

## Ethics Statement

The animal study was reviewed and approved by Comité d’Ethique pour l’Expérimentation Animale Secteur des Sciences de la Santé UCLouvain (2011/UCL/MD/015 and 2015/UCL/MD/015).

## Author Contributions

Conception and design: DH and BV. Methodology: DH and BV. Acquisition of data: DH, TD, VS, and FS. Writing: DH and BV. Study supervision: BV. All authors contributed to the article and approved the submitted version.

## Funding

This work was supported by the Ludwig Institute for Cancer Research, the Fonds Scientifique pour la Recherche–FNRS, de Duve Institute and UCLouvain (Belgium). Financial support for authors: DH, FNRS-FRIA (Grant number: 1.E082.14); TD, FNRS-Télévie (Grant number: 7.4597.18) F. Schramme: UCLouvain; VS: Ludwig Institute for Cancer Research; BV: Ludwig Institute for Cancer Research.

## Conflict of Interest

BV is a co-founder of iTeos Therapeutics.

The remaining authors declare that the research was conducted in the absence of any commercial or financial relationships that could be construed as a potential conflict of interest.
