# Supplementary material for: Tryptophan 2,3-Dioxygenase Expression Identified in Murine Decidual Stromal Cells Is Not Essential for Feto-Maternal Tolerance
Source: Front Immunol. 2020 Dec 8;11:601759. doi: 10.3389/fimmu.2020.601759 (PMC7752949; doi:10.3389/fimmu.2020.601759)
Supplement: Supplementary file 1 [file DataSheet_1.pdf]

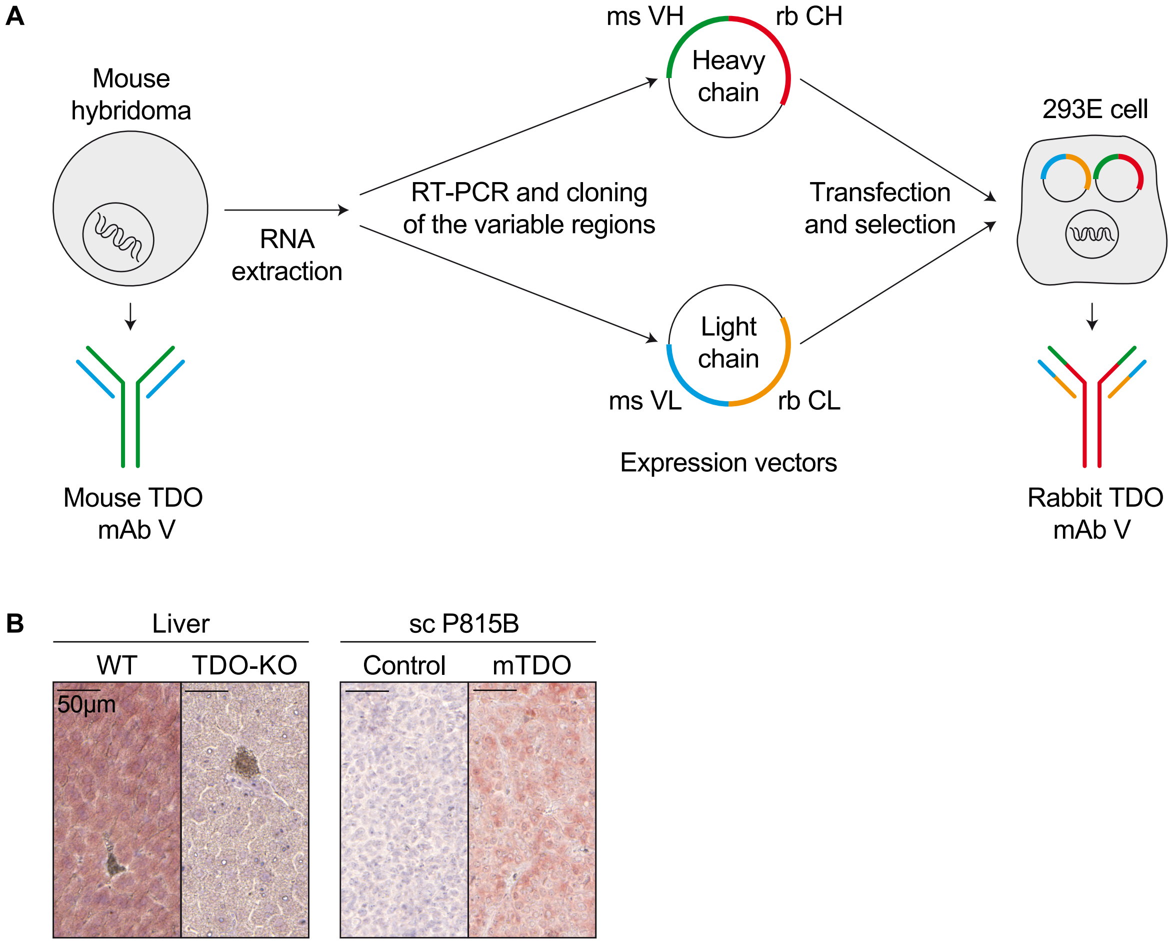

Supplementary figure 1. Production and validation of the chimeric rabbit TDO mAb V antibody.

(A) RNA was extracted from the mouse hybridoma producing the TDO mAb V antibody. The variable regions of the heavy and light chains were amplified with specifically selected primers and cloned in expression vectors containing the constant regions from rabbit heavy and light IgG chains in the correct reading frame. 293E cells were transfected and selected with zeocin and blasticidin. The antibody was then purified from the cell culture supernatant. The mouse heavy chain is stained in green, the mouse light chain in blue, the rabbit heavy chain in red and the rabbit light chain in orange.

(B) TDO was revealed by IHC using the chimeric TDO mAb V on mouse FFPE tissue sections of WT liver, TDO-KO liver and subcutaneous (sc) P815B tumors expressing or not mTDO upon transfection. Negative controls were performed by omitting the primary antibody and remained unstained.

Abbreviations: mAb = monoclonal antibody; FFPE = formalin-fixed, paraffin-embedded; ms = mouse; rb = rabbit; CL = constant light chain; CH = constant heavy chain; VL = variable light chain; VH = variable heavy chain.
