# Supplementary material for: Tryptophan 2,3-Dioxygenase Expression Identified in Murine Decidual Stromal Cells Is Not Essential for Feto-Maternal Tolerance
Source: Front Immunol. 2020 Dec 8;11:601759. doi: 10.3389/fimmu.2020.601759 (PMC7752949; doi:10.3389/fimmu.2020.601759)
Supplement: Supplementary file 2 [file DataSheet_2.pdf]

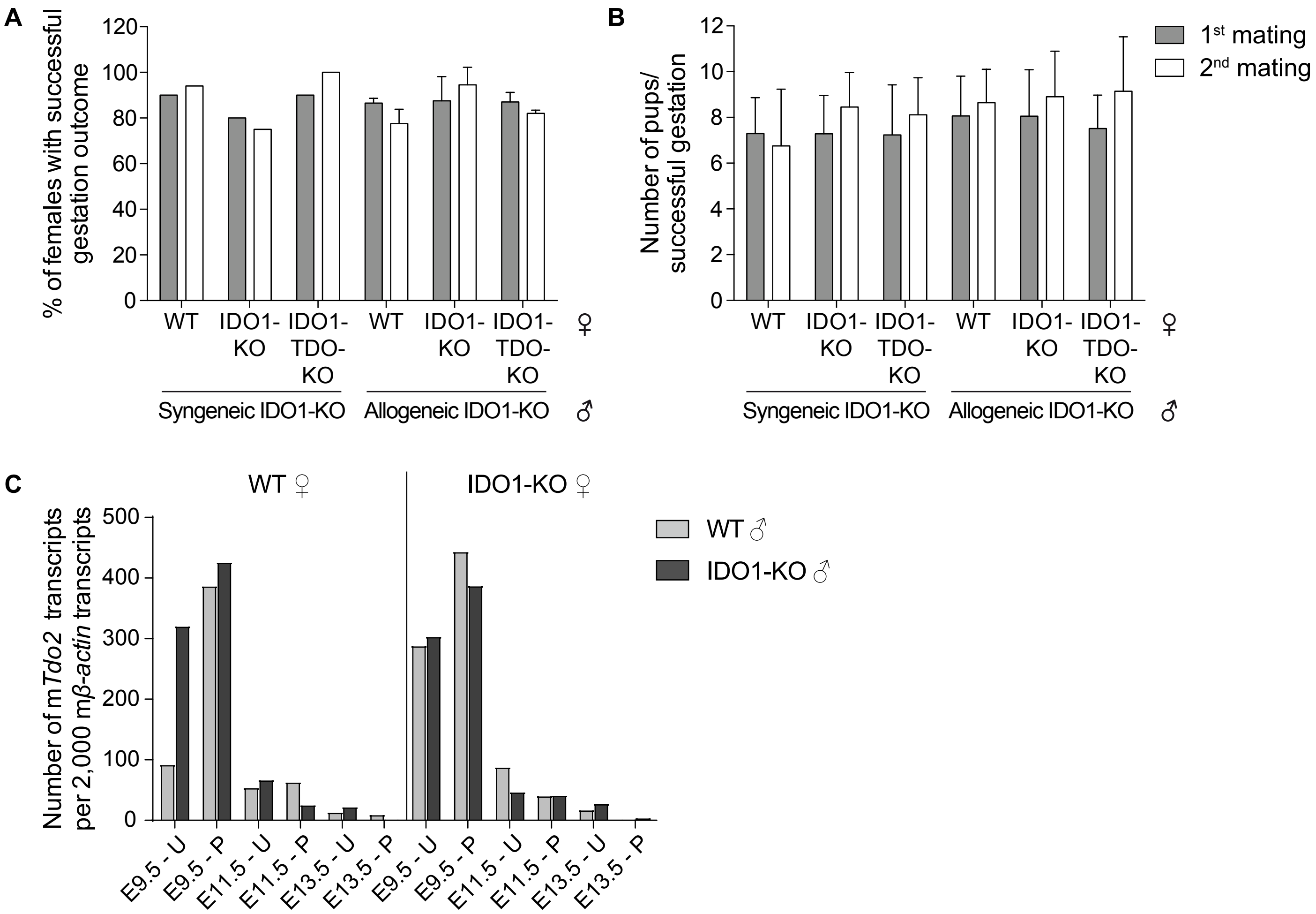

Supplementary figure 2. Outcome of gestation in IDO1-KO and double IDO1-TDO-KO females.

WT littermate, IDO1-KO littermate or IDO1-TDO-KO B/6 females were mated with either syngeneic IDO1-KO B/6 males or allogeneic IDO1-KO BALB/c males (20 females/group). The number of females giving birth and the number of pups per successful gestation were counted (grey bars). The females that gave birth were mated a second time with the same males (white bars).

(A) Percentage of females giving birth (mean ± SD of 1 (syngeneic) or 2 (allogeneic) independent experiments).

(B) Numbers of pups per successful gestation (mean ± SD of pups pooled from 1 (syngeneic) or 2 (allogeneic) independent experiments).

(C) WT and IDO1-KO B/6 females were mated with syngeneic WT or IDO1-KO B/6 males and RNA was extracted separately from placenta and uteri between days E9.5 and E13.5 of gestation (the same samples as in Figure 5A). *Tdo2* expression was measured by RT-qPCR and reported to 2,000 copies of murine β-actin (mean ± SD).
