# Supplementary material for: Tryptophan 2,3-Dioxygenase Expression Identified in Murine Decidual Stromal Cells Is Not Essential for Feto-Maternal Tolerance
Source: Front Immunol. 2020 Dec 8;11:601759. doi: 10.3389/fimmu.2020.601759 (PMC7752949; doi:10.3389/fimmu.2020.601759)
Supplement: Supplementary file 3 [file DataSheet_3.pdf]

A

Genotypes	Mean No. of concepti per ♀ (No. of females with concepti / No. of ♀ treated)	
	**1MT**	**Placebo**
♀ CBA × ♂ CBA	4.0 ± 2.5 (4 / 9)	4.67 ± 2.1 (3 / 8)
♀ CBA × ♂ B/6	6.9 ± 2.0 (7 / 9)	6.3 ± 2.5 (3 / 8)

B

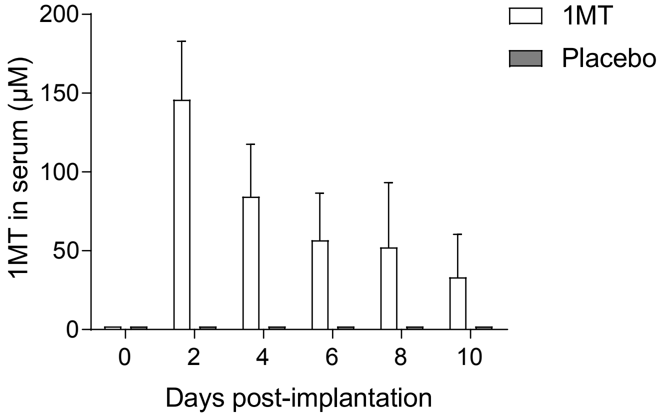

Supplementary figure 3. Effect of the IDO1 inhibitor 1MT on maternal fertility.

(A) WT CBA females were mated with either syngeneic CBA males or allogeneic B/6 males. On embryonic age E4.5, females were anesthesized with isoflurane and 10-day release pellets with 200 mg of 1-methyl-DL-tryptophan (1MT) or corresponding placebo pellets (purchased from Innovative Research of America) were implanted subcutaneously in the back of the female. Concepti were counted on E15.5. The table lists the mean number of concepti per female ± SD (excluding females without any concepti). No statistically significant difference was observed betweed 1MT- and placebo-treated females (using an unpaired Mann-Whitney test). Between brackets are listed the number of females with concepti on E15.5 and the total number of females treated with 1MT or placebo pellets.

(B) The serum concentration of 1MT was measured in non-pregnant mice because bleeding is a stressful procedure that may have a negative impact on pregnancy outcome. Pellets were implanted in non-pregnant females (5 females per group) and blood was taken by tail vein bleeding every two days. Serum was collected and 1MT was measured by HPLC (mean ± SD). Control bleeding (day 0) was performed just before surgery.
